# The Implication of New Developments in Hemophilia Treatment on Its Laboratory Evaluation

**DOI:** 10.7759/cureus.30212

**Published:** 2022-10-12

**Authors:** Garima Anandani, Tarang Patel, Riddhi Parmar

**Affiliations:** 1 Pathology, All India Institute of Medical Sciences, Rajkot, Rajkot, IND

**Keywords:** factor viii equivalence, non-factor therapies, thrombin generation test, chromogenic assay, one-stage clotting assay

## Abstract

Laboratories monitor hemophilia replacement therapy by specific coagulation factor measurement before and after the infusion of human-derived or recombinant factors. Bypassing agents are now used for patients with inhibitors. Recently, modified long-acting coagulation factors have been introduced, for which discrepant results may be expected when the measurement is performed with one-stage clotting or chromogenic assays. Currently, novel drugs not based on coagulation factors are being developed and further tested in clinical studies. These drugs do require new methods, and therefore, laboratory evaluation of hemophilia will undergo dramatic changes in the near future. Accordingly, present laboratory methods for monitoring, which include one-stage clotting or chromogenic assays, used to measure either factor VIII (FVIII) or factor IX (FIX), will not be sufficient. A thrombin generation test (TGT) or thromboelastometry may be used to monitor bypassing agents. For measuring modified long-acting coagulation factors, chromogenic assays will be probably more suitable than one-stage clotting assays. Novel drugs that are not based on coagulation factors, such as emicizumab, fitusiran, or concizumab, will require alternative methods.

## Introduction and background

Hemophilia A and B are X-linked disorders of coagulation having factor VIII (FVIII) deficiency and factor IX (FIX) deficiency, respectively. The prevalence of hemophilia A is one case per 5,000 males, and that of hemophilia B is one case for every 30,000 males [1]. The treatment of hemophilia comprised of either fresh frozen plasma or cryoprecipitate concentrates, which advanced to high-purity, plasma-derived FVIII/FIX concentrates and recombinant FVIII/FIX concentrates during the last 50 years [2].

FVIII-specific activated partial thromboplastin time (aPTT)-based one-stage clotting assays or two-stage chromogenic assays are used for laboratory monitoring of FVIII replacement therapy [3]. The recombinant B-domainless FVIII concentrates showed an assay discrepancy, in which levels measured using a one-stage clotting assay were 20%-50% lesser than that by a chromogenic assay [4].

Modified FVIII molecules were prepared by the attachment of polyethylene glycol, fusion to the Fc portion of immunoglobulin (Ig) G, or various other types of modifications that change the physical characteristics of the FVIII molecule. They have an extended half-life and also show assay discrepancy due to altered behavior in various assays [5]. These concentrates are neutralized in patients who develop high-titer inhibitors. Hence, bypassing agents such as activated prothrombin complex concentrate (APCC), also known as factor 8 bypassing agent (FEIBA), containing factor II, VIIa, IX, and X, or activated recombinant FVII (rFVIIa) are used to treat such patients [6,7]. A thrombin generation test (TGT) or thromboelastometry can be used to monitor these bypassing agents [8].

Along with FVIII molecules, FVIII gene therapy can also be used now as replacement therapy. Recently, non-factor therapies comprising novel drugs such as emicizumab, the bispecific antibody; fitusiran, a small interfering RNA-based molecule that reduces the expression of antithrombin (AT); and concizumab, antibodies blocking the activity of tissue factor pathway inhibitor (TFPI) are available or are in clinical trials. These newer treatment modalities need updated methods for laboratory monitoring [9]. Herein, we will focus on the various testing methods, lacunae in the current testing methodology along with the analytic and post-analytic issues involved, and recent advances in the newer testing modalities.

## Review

Hemophilia is diagnosed clinically based on repeated episodes of bleeding since childhood. These may start from the neonatal period in the most severe forms and are confirmed on laboratory testing [8].

Types of assays

One-Stage and Two-Stage Chromogenic Assays

**Figure 1:**
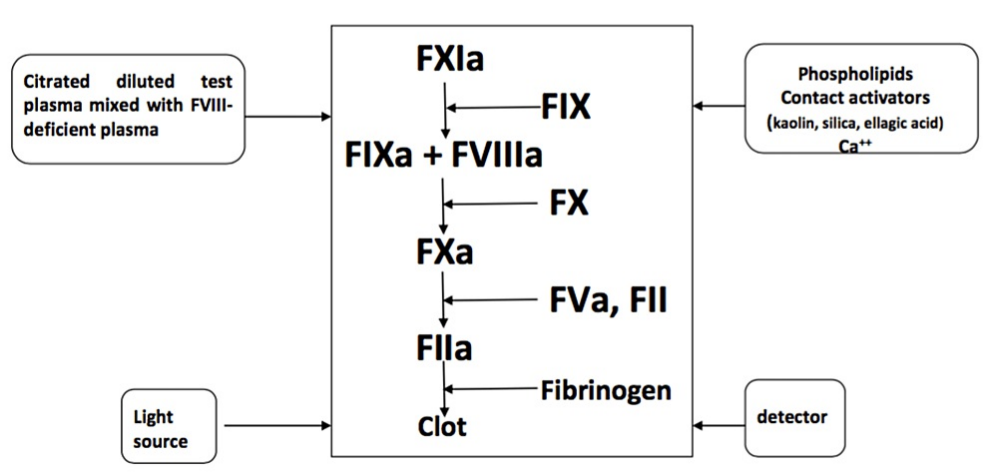
Principle of the one-stage aPTT-based clotting assay for FVIII.  For measuring FIX levels, FIX-deficient plasma is used instead of FVIII-deficient plasma. aPTT: activated partial thromboplastin time, FVIII: factor VIII, FIX: factor IX

Factor VIII:C levels are usually measured by an aPTT-based one-stage assay throughout the world for the last five decades. It is based on the principle that a control sample containing factor VIII corrects the prolonged aPTT or delayed clotting of plasma with FVIII deficiency [10]. The patient’s plasma is mixed with FVIII-deficient plasma, in the presence of an activating agent (surface activator such as kaolin, micronized silica, or ellagic acid) and calcium, which activates the coagulation pathway (Figure 1) [11]. The patient’s FVIII activity should be reported as the normal percentage activity compared with control plasma, which should be run simultaneously under similar conditions.

This test assay results are highly fluctuating due to the variability in contact factor activators or phospholipids. Factor-deficient plasma is also responsible for these varying results due to its multiple sources, the technique of its preparation, residual factor activity, and optimization of other factors such as von Willebrand factor (vWF) activity [8]. However, a normal FVIII:C level measured by a one-stage assay does not rule out mild hemophilia A [10].

**Figure 2:**
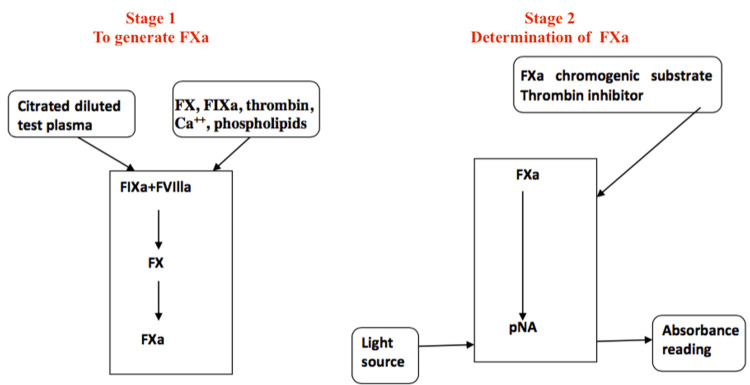
Principle of the two-stage or chromogenic assays for FVIII. By minor modifications of reagents, FIX can also be measured by the same principle. FVIII: factor VIII, FIX: factor IX, pNA: para-nitroaniline chromogen

In the two-stage or chromogenic assay, the diluted patient’s plasma is mixed with purified factor X, IXa, calcium, phospholipids, and thrombin-producing FXa, which is quantified by hydrolyzing a substrate (Figure 2) [12]. These assay results are less variable as factor-deficient plasma is not needed and lupus anticoagulants do not interfere much with the test results. It has a higher cost per test than the one-stage clotting assay (Table 1). Preparing aliquots and freezing reagents after reconstitution, and testing many samples together in batches can make it more cost-effective [13].

**Table 1:** Major characteristics of one-stage assay and chromogenic assay.

One-stage clotting assay	Chromogenic assay
Familiar and widely available	Lack of familiarity
Relatively cheap	Expensive
Clinical experience	Relatively poor clinical experience
Relatively poor reproducibility	Acceptable reproducibility
Need for factor-deficient plasma	No need for factor-deficient plasma
Relatively large between-reagent variability	Relatively insensitive to interference from lupus anticoagulants
	Suitable to be run in emergency situations

Few cases of mild hemophilia A have discrepancies in the test results of FVIII activity obtained by both the abovementioned assay types [14]. These may be due to different mutations in the FVIII or FIX gene, leading to different assay outcomes [15]. The availability of various types of treatment modalities may also lead to discrepancies during the monitoring of therapy, for example, while using long-acting FVIII or FIX concentrates [8].

The most common type of deviation is that the test results of the one-stage assay are higher than that of the chromogenic assay. However, in 75% of these cases, the results are less than the normal reference range, and so, the case is reliably diagnosed as hemophilia by both tests. However, in a few cases, one-stage assay test results are normal, while they are reduced by chromogenic assay. There is a history of bleeding, and genetic mutations are also identified in many of these cases, which correlate with the reduced factor levels seen in chromogenic assay testing [16]. About 5%-10% of mild hemophilia A cases have normal aPTT and normal FVIII activity levels by one-stage assay. On the other hand, a few cases of mild hemophilia A have lower FVIII activity levels by one-stage assay but normal by chromogenic assay. These patients have no personal or family history of bleeding and clinically do not require therapy for hemophilia, hence again correlating with the result obtained by chromogenic assay [17].

Therefore, all hemophilia centers are recommended to make availability of and perform a chromogenic assay in patients with normal aPTT and one-stage FVIII activity levels but a positive personal or family history of bleeding.

Thrombin Generation Testing and Thromboelastometry

**Figure 3:**
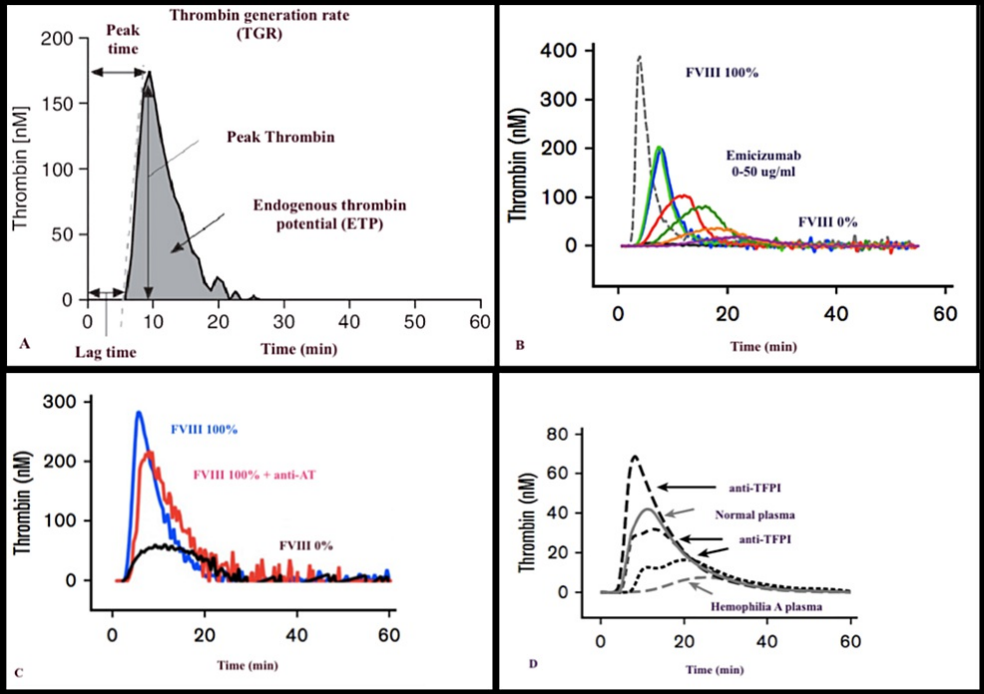
Thrombin generation curve and its parameters (A). Thrombin generation in a FVIII-deficient plasma in the presence of emicizumab (B), anti-AT (C), and anti-TFPI antibodies (D). FVIII: factor VIII, AT: antithrombin, TFPI: tissue factor pathway inhibitor

Global coagulation assays such as the thrombin generation test (TGT) correlate well with bleeding tendency but have neither been used as a screening test nor to classify disease severity. The former part of a normal thrombin generation curve is due to FVIIa/TF producing FIXa and FXa, along with the short-term availability of the labile FVIIIa, which lasts for a few minutes, whereas the latter part of the curve is due to AT-mediated inhibition of FIXa, FXa, and thrombin (Figure 3).

FVIII or FIX activity and most of the TGT parameters were found to be significantly correlated statistically, but this coefficient of correlation was not optimal, which suggests that many other parameters were also evaluated by the global coagulation assays [10].

Cases having FXI deficiency usually are mild bleeders. However, patients having similar levels of FXI activity can present clinically with variable bleeding tendencies. Routine laboratory tests diagnose FXI deficiency, but not the risk of bleeding, while TGT can differentiate bleeders from non-bleeders, as the thrombin peak and velocity are severely hampered in cases manifesting severe bleeding clinically, irrespective of the FXI levels. TGT or whole blood thromboelastometry can be used to predict the response to bypassing agents by testing before and after in vivo administration because probably a specific thrombin level produced determines the effectiveness of the drug clinically. TGT may also help in the dose optimization of bypassing agents before and after operative procedures or in life-threatening situations and also in preparing their customized effective infusion schedule in all patients [8,10].

Newer treatment modalities

These treatment modalities include newer replacement as well as non-replacement therapies.

Long-Acting Coagulation Factor Concentrates

Human-derived or recombinant FVIII and FIX have short half-lives ranging from eight to 12 hours for FVIII and from 18 to 24 hours for FIX in vivo, due to which repeated frequent concentrate infusion is required in hemophiliacs [18]. Products with relatively longer half-lives have been introduced [19,20].

Newer recombinant factor concentrates conjugated with polyethylene glycol, albumin, or the Fc fraction of Ig have been produced, which have significantly longer half-lives in vivo with similar clinical efficacy. FIX half-life is successfully prolonged significantly to five days, while that of FVIII to two days. This difference is because FVIII is bound to vWF in blood circulation and both their half-lives are related to each other. Therefore, modification of vWF would also be needed to further prolong the half-life of FVIII.

Measuring their levels is significantly discrepant mainly because, in one-stage and two-stage assays, a standard plasma is mandatory to be run along with the patient plasma for calculating the factor activity levels. This standard plasma is usually pooled from the plasma of healthy human donors having native or unmodified FVIII or FIX. However, here, the test plasma from the patient contains modified coagulation factors. Hence, there is a discrepancy in the assays in testing the modified and native factors, which are recognized variably. These discrepancies were minimized for recombinant antihemophilic factor for hemophilia A (ReFacto) when measured post-infusion if an aliquot of the same concentration was used at certified fixed potency same as the standard [21].

A long-acting modified recombinant FIX showed a discrepancy in one-stage assay when reagents having silica as an activator resulted in higher FIX activity, while those having ellagic acid as an activator resulted in factor levels similar to that by chromogenic assay [22]. Long-acting modified recombinant FIX adheres to the surface of silica, unlike ellagic acid, leading to increased activation of coagulation and overestimation of factor activity [23].

Clinically, patients might be overt- or under-treated based on the test assay used for monitoring. The US National Hemophilia Foundation recommends that chromogenic assays should be used to investigate hemophilia patients; however, they are not yet approved by the FDA [24].

Non-replacement Therapy to Treat Hemophilia

Bypassing agents: These include APCC and rFVIIa, which are used in hemophiliacs with high titer inhibitors [6]. APCC act through potentiation of coagulation through the optimization of prothrombin complex factors, and rFVIIa directly activate the coagulation cascade. Global coagulation tests can be used to monitor them [7]. APCC increased the thrombotic risk as a few patients on APCC developed thrombotic microangiopathy or venous thromboembolism [8].

Non-factor therapies: Therapy with these drugs does not need coagulation factor infusions and is introduced especially for prophylaxis of hemophiliacs [8].

Emicizumab is a bispecific monoclonal human antibody that mimics FVIIIa activity. It comprises two antigen-binding domains, one of which binds to FIX/FIXa and the other binds to FX/FXa. Hence, it bridges FIXa and FX, increasing FX activation mediated by FIXa, producing more FXa, and increasing thrombin formation, leading to highly improved hemostasis [25]. There was a significant reduction in bleeding episodes in cases of hemophilia A on emicizumab therapy, irrespective of the presence or absence of inhibitors [26,27]. In approximately 33% of cases on emicizumab prophylactic treatment, bypassing agents were needed to control breakthrough bleeding [8].

Fitusiran is an RNA interference therapeutic agent that acts on the antithrombin (AT) gene in hepatocytes. N-Acetylgalactosamine (GalNAc)-tagged oligonucleotide binds and degrades messenger RNA-AT, leading to posttranscriptional silencing of the AT gene expression and hence prevention of the AT synthesis [28,29]. Reduction in AT levels leads to increased availability of thrombin, FXa, and FIXa, which increases hemostasis even in the absence of FVIII. Most of the cases on fitusiran are reported to show a reduced bleeding tendency; however, one patient was reported to have a fatal thrombotic event [30,31].

Concizumab is one more inventive application based on the alteration of the tissue factor pathway inhibitor (TFPI) activity by the presence of a humanized monoclonal anti-TFPI antibody [8]. Concizumab, marstacimab, BAY-1093884, and MG1113 are four novel drugs acting as monoclonal anti-TFPI antibodies. TFPI inhibits FXa and factor VIIa/tissue factor (FVIIa/TF) complex. This is counteracted by these drugs, leading to increased availability of the FVIIa/TF complex, which produces a larger amount of FIXa and FXa, ultimately increasing the production of thrombin and reduced bleeding tendencies in hemophiliacs. However, few cases of thrombotic events have been reported [32,33].

**Figure 4:**
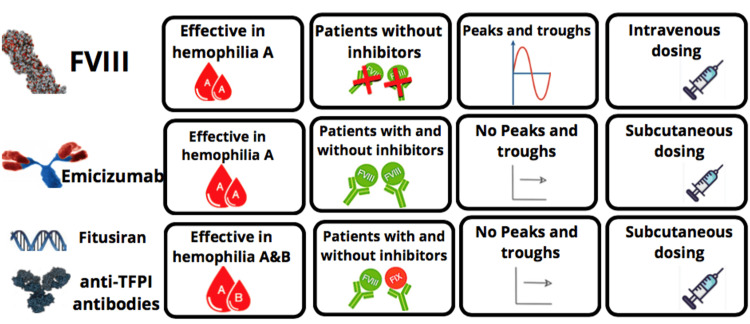
Differentiating characteristics in the indication, route of administration, and therapeutic levels of FVIII replacement therapy, emicizumab, fitusiran, and anti-TFPI antibodies. FVIII: factor VIII, TFPI: tissue factor pathway inhibitor

Frequent intravenous infusions mostly thrice a week are required for replacement therapy with FVIII concentrates. FVIII reaches its peak levels post-infusion while decreases gradually until the next infusion. However, in non-factor therapies, there are subcutaneous administrations of the drug, peak and trough are absent, and levels of the novel drug are stabilized (Figure 4).

These differential features have a prominent influence on how patients on these non-factor therapies will be monitored [5].

Gene Therapy

Innovations have been made in gene therapy in the past decade, and Roctavian is the first gene therapy used for treating hemophilia A. Adeno-associated virus (AAV) is modified not to produce disease in humans. It acts as a vector and has a wild-type or modified FVIII or FIX gene, which helps in achieving stable FIX or FVIII expressions, increasing the factor levels, and improving clinical bleeding phenotype. Genome editing using clustered regularly interspaced short palindromic repeats (CRISPR/Cas9) method, which is still in the experimental phase, is a promising technology to treat hemophilia [1]. However, there are challenges in the laboratory monitoring of these patients, as the FVIII and FIX that are produced after gene therapy have variable characteristics and so are differently identified by various assays [8].

Laboratory monitoring of non-factor therapies

In patients receiving non-factor therapy, we need to monitor the levels of therapeutic agents, their effect on the homeostasis of the patient, and their FVIII equivalence.

Monitoring of Non-factor Therapeutic Drug Concentration

The clinician has to decide how frequently non-factor therapeutic drugs should be monitored in every patient. Each non-factor drug will require a different type of testing technique [5].

Many methods are available to measure emicizumab levels, of which an enzyme-linked immunosorbent assay (ELISA)-based assay using anti-idiotype antibodies was used in clinical trials, although not available commercially [34]. Prothrombin time (PT) and thrombin time (TT) are not influenced by emicizumab. Tests based on the activation of the intrinsic pathway of coagulation are affected, such as activated coagulation time and aPTT-based activated protein C resistance assay [8]. FVIII activity assays can be used to monitor emicizumab levels as it somewhat mimics the FVIII cofactor function [5]. However, it normalizes the aPTT at much lower levels than what is seen after subcutaneous injection of clinically effective doses in vivo [35]. Hence, there is no utility of a one-stage aPTT-based assay. A modified aPTT assay can be used wherein the plasma samples are further sequentially diluted to see linearity between emicizumab concentrations and aPTT [36,37].

Another option is chromogenic assays for FVIII activity testing, whereby a near-linear relationship is established between emicizumab concentrations and FXa production [38]. Distinct emicizumab calibrators are available for both these test assays. The results obtained by these assays depict FVIII-like activities and are not true FVIII equivalence [39]. The activity of emicizumab depends on the accessible FIXa levels, which are variables pertaining to the different activating compounds in the aPTT reagent. FIXa levels also depend on TF or FXIa concentrations. These results are also related to the availability of other procoagulant molecules such as FVIII or APCC in the plasma samples containing emicizumab [38]. One more challenge is assessing FVIII inhibitor titers in a patient on emicizumab who may also need therapy with bypassing agents. As emicizumab will affect the traditional method of measuring inhibitor titer, a chromogenic assay using bovine reagents may be used for the same, which are completely insensitive to emicizumab [8].

For fitusiran, the residual oligonucleotide levels in the hepatocytes of the patients cannot be practically measured. So, we can quantify residual AT levels using antigen assays or thrombin- or FXa-based activity assays. Here, also, concurrent therapy with APCC might modify the results of these activity assays [40].

There are no distinct tests available yet for measuring anti-TFPI antibody levels. Therefore, antigen TFPI levels should be measured using ELISA. However, there is no confirmation whether all the available anti-TFPI antibody drugs actually reduce plasma TFPI levels or if these monoclonal antibodies interfere with testing by ELISA. Another option available is to measure residual TFPI activity using specific activity assays, including a diluted PT-based assay and TF-dependent chromogenic assays [41,42]. It should be taken into consideration that only 10%-30% of total TFPI in the body is present in plasma [43]. The amount of TFPI inhibited at the vascular lining cannot be identified by these assays.

Hence, direct measurement of emicizumab, fitusiran, or any anti-TFPI antibody is cumbersome, but indirect activity assays can be used to monitor their drug concentration and activity [5].

Monitoring the Therapeutic Efficacy of Non-factor Drugs on Patient Hemostasis

TGT, thromboelastography, and clot waveform analysis should be performed before and after starting the treatment with non-factor drugs for analyzing their effect by observing the changes in the patient’s hemostatic potential [44]. In TGT, the presence of the emicizumab or the functional absence of AT or TFPI leads to increased thrombin peaks and the endogenous potential of thrombin generation in comparison to only FVIII-deficient plasma (Figure 3). This correlates with the accentuated hemostasis caused by these drugs.

However, data should be interpreted carefully as the correlation between an individual’s thrombin generation potential in vitro and the actual true hemostatic response in a patient is still ambiguous. So, whether these assays can correctly anticipate the clinical outcome is yet not proven [45,46]. Also, these global tests do not depict the true FVIII equivalence of the agent. TGT can also be utilized for analyzing the outcome of add-on therapeutic agents such as FVIII or APCC, even before they are given to the patient [47].

Monitoring the FVIII Equivalent Levels of Non-factor Therapeutic Agents

Due to an extensive and prolonged clinical experience with the use of FVIII replacement therapy, the FVIII levels required for distinct types of activities or surgeries have been successfully accomplished. Therefore, to identify the extent to which the non-factor agents will defend against bleeding during such conditions, it will be appropriate to know their FVIII equivalence. The mechanism of action for every non-factor therapeutic agent is essentially disparate compared to that of FVIII. In emicizumab therapy, FIXa is the limiting factor. Hence, the true FVIII equivalence for emicizumab cannot be established as it is FIXa-dependent. Animal models can provide a better and impartial response whereby injuring an animal activates natural procoagulant and anticoagulant pathways.

Kitazawa et al. used the primate model, which was the first animal model to obtain FVIII equivalence for emicizumab in acquired hemophilia A. Anti-FVIII antibodies were injected in primates, which inhibited their FVIII, followed by either emicizumab (6 mg/mL) or porcine FVIII (0.01 U/mL, which cannot be detected by anti-FVIII antibody). Blood loss was prevented by both of these, which was measured by hemoglobin levels; however, porcine FVIII was slightly more efficient than emicizumab [48]. Finally, the authors concluded that the activity of emicizumab at this concentration corresponds to about 1% FVIII activity. At present, the therapeutic dose of emicizumab used is 55 mg/mL, which is 10 times higher than the primate model, and hence, accordingly, its FVIII equivalence is around 10% [49]. An adapted mouse model was established wherein FVIII deficiency was combined with the availability of human FIX and FX, in which the therapeutic dosage of emicizumab was found to be equivalent to 9% of FVIII levels, which was almost similar to the primate model, wherein the estimated level was 10% [50]. These FVIII equivalent levels in animals and humans depend upon the type, severity, and location of injury inflicted, which will decide the amount of FIXa production. This generation of FIXa is related to the levels of TF or FXIa present following the injury and will be required for the action of emicizumab [5].

For identifying the in vivo factor VIII equivalence of fitusiran, a saphenous vein bleeding model was prepared [28]. Mice with FVIII deficiency were given either fitusiran, which reduced AT antigen by 70% in vivo, or 25 U/kg of FVIII, which results in 0.5 U/mL or 50% FVIII levels in vivo. Herein, AT reduction turned out to be as efficient as by FVIII. Recently, a tail clip model has been developed wherein nanobodies are used to block AT activity in FVIII-deficient mice [51]. Based on the outcome of these research models, it is concluded that when AT levels are reduced to 30% of normal, their efficiency is equivalent to 20% of FVIII levels.

A cuticle bleeding model was developed to study the effect of concizumab [52]. FVIII-deficient rabbits were treated with a dose range of 0.5-8 mg/kg. After 35 minutes of drug administration, bleeding was induced. The drug dose of 0.5 mg/kg was effective enough to reduce the bleeding tendency markedly. A dosage of 1 mg/kg decreased the loss of blood to an extent similar to that seen normally in rabbits without hemophilia. The anti-TFPI levels in plasma were however not reported. As plasma volume in rabbits is 40 mL/kg and supposed availability is 90%, after a 1 mg/kg IV injection, the antibody levels would be approximately 22 mg/mL. Due to a pronounced attrition in bleeding tendency and severity, it was presumed to have an FVIII equivalence of at least 20%.

The interpretation of these research models has a major drawback of unknown interspecies differences that can probably affect the effectiveness of these non-factor therapeutic agents. Also, there was an absence of FVIII calibrators in the research studies for evaluating fitusiran and concizumab [5].

## Conclusions

Various types of FVIII concentrates are available, such as plasma-derived or recombinant and short-acting or long-acting, and they may be tested from the patient’s plasma or directly from concentrates. Because there are numerous accessible options, the laboratory analysis of FVIII levels possesses a lot of challenges. For post-infusion monitoring of long-acting coagulation factor concentrates, a chromogenic assay should be performed instead of a one-stage assay. Standards specific to products tested or the same as the tested product should be used for better results. Bypassing agents are used to treat hemophiliacs with the development of inhibitors that can be monitored post-infusion by global coagulation tests such as TGT or thromboelastometry.

The advent of non-factor, non-replacement therapeutic agents such as emicizumab, fitusiran, and concizumab has increased these difficulties even more. ELISA-based assay, aPTT-based activated protein C resistance assay, or FVIII activity chromogenic assays can be used for monitoring emicizumab levels. For fitusiran, residual AT levels can be quantified using antigen assays or thrombin- or FXa-based activity assays. Anti-TFPI antibody drugs can be monitored by measuring antigen TFPI levels by ELISA or residual TFPI activity using specific activity assays such as a diluted PT-based assay and TF-dependent chromogenic assays. TGT, thromboelastography, and clot waveform analysis can be used to observe the effect of these non-factor drugs on the hemostatic potential of the patient.

Complete knowledge of the biochemical composition and actions of these novel treatment options is mandatory for the proper selection of tests and interpretation of results. It is also important to know about various inter-assay and inter-therapy discrepancies that can be seen. The laboratory personnel should be preinformed of the patient-specific treatment given so that they can decide about which test to be performed correctly. Clinical laboratories should have all the facilities to perform various types of assays, which are required for monitoring the therapeutic drug levels and the response of the patient to therapy.
